# Vibration velocity and frequency characteristics of surrounding rock of adjacent tunnel under blasting excavation

**DOI:** 10.1038/s41598-022-12203-7

**Published:** 2022-05-19

**Authors:** Yi Luo, Hangli Gong, Dengxing Qu, Xueping Zhang, Yuhang Tao, Xinping Li

**Affiliations:** 1 Hubei Key Laboratory of Roadway Bridge and Structure Engineering, Wuhan University of Technology, Wuhan, 430070 China; 2 School of Civil Engineering and Architecture, Wuhan University of Technology, Wuhan, 430070 China; 3 Sanya Science and Education Innovation Park, Wuhan University of Technology, Sanya, 572024 China

**Keywords:** Engineering, Civil engineering

## Abstract

The aim of this study is to improve the accuracy of response prediction and safety evaluation of blasting vibration of a deeply-buried tunnel group. For this purpose, the expression of frequency-domain and blasting vibration velocity spectra for the equivalent blasting load in multiple holes was derived through theoretical analysis, and propagation and attenuation of the primary frequency of blasting vibration of multiple cutting holes and caving holes in the infinite rock mass were explored. Response characteristics of vibration frequency spectra in rock surrounding of the adjacent tunnel induced by full-section blasting excavation of the tunnel under the high in situ stress were studied using the dynamic finite element method. The research indicates that blasting vibration waves have the greatest influences on the adjacent tunnel at the haunch in the side facing the blasting, where the vibration velocity is inversely proportional to the spacing between tunnels and directly proportional to the tunnel diameter. The centroid frequency increases with the increase of the spacing between tunnels and tunnel diameter. Furthermore, vibration velocity spectra at the most affected location (namely the haunch) in the side facing blasting of the adjacent tunnel under different conditions were derived. The coincidence of the theoretical formula was verified by comparing measured data of blasting of diversion tunnels in Pubugou Hydropower Station (Sichuan Province, China). The research results can provide theoretical guidance and reference for the prediction of blasting vibration response of similar projects in the future.

## Introduction

Drilling and blasting method is the most widely used and efficient method for excavation of deep rock masses in engineering practice^1^. With the continuous expansion of the design scale of underground powerhouse caverns, the problems of loosening and deformation of rock surrounding induced by blasting excavation of rock mass under the high in-situ stress in deep parts are particularly prominent^2^. The control and prediction of blasting vibration are also common difficulties encountered in engineering practice^3^. Previous studies have paid less attention to frequency spectral characteristics and attenuation of blasting vibration and the existing attenuation formulae for the primary frequency can only be used in specific cases. So far, there is no attenuation formula for the primary frequency generally accepted by scholars^4^, and there is a lack of research into the influences of the spatial distribution of underground tunnels on characteristics of vibration frequency spectra in the surrounding rock.

Since the 1920s, Rudenk^5^ and Tedesco et al.^6^ have studied propagation and influence factors of blasting vibration waves in surrounding rock as well as effects of blasting vibration on adjacent buildings (structures). In general, a single parameter is used to describe safety criteria of blasting vibration, mainly including velocity, displacement, and acceleration, and the three quantities can be interchanged and converted. The peak vibration velocity of particles has been widely used to evaluate the intensity of blasting vibration. Sadovski’s formula considers factors, such as the amount of explosive and distance from blasting center, which is close to the actual results and is widely used in prediction and control of blasting vibration^7–9^. In recent years, many scholars have researched propagation and attenuation of blasting vibration waves in the surrounding rock. Resende et al.^10^ highlighted the importance of clarifying stress wave propagation paths and local peak value in control of blasting vibration. Based on three-dimensional distinct element code (3DEC) simulation, Babanouri et al.^11^ studied propagation of blasting waves in fractured rock mass. By virtue of a model test, Dong et al.^12^ researched propagation and attenuation of cylindrical blasting-induced stress waves in jointed rock mass under different initial stresses. Kim et al.^13^ studied the influences of direction and spacing between joints in rock surrounding on propagation of blasting vibration waves. Sadique et al.^14^ studied the vibration response of tunnels with three common rock types (granite, basalt and quartzite) under blasting vibration. Ding et al.^15^ discussed the role of safety monitoring considering effects of tunnels in evaluation on the stability of a rock mass. Based on finite element analysis, Bumjoo et al.^16^ explored the stability of tunnels and rock columns during the expansion of existing parallel tunnels. Xia et al.^17^ investigated influences of blasting excavation of tunnels on the surrounding rock and lining structure of adjacent tunnels through an in-situ test. Fei et al.^18^ established a relationship between the blasting vibration-induced disturbance and adjacent tunnels by means of the acoustic testing technique. Zaid et al.^19,20^ studied non-linear finite element analysis of rock tunnel having shear zone subjected to internal blast loading, and established the damage relationship of shallow unlined tunnel under TNT blast load.

As research on blasting vibration improves, scholars have found that there are limitations and shortcomings when using the vibration velocity alone as the safety criterion of blasting vibration in a lot of practical projects. They stated that influence factors for effects of blasting vibration should consider influences of vibration frequency and duration. Therefore, scholars have analyzed frequency spectra of blasting vibration signals based on methods, such as Fourier transform, wavelet transform, and Hilbert–Huang transform (HHT) analysis^21,22^. Yang et al.^4,23^ studied the Peak Particle Velocity (PPV) attenuation and frequency characteristics for the rock vibration induced by transient stress release and its combined actions with blast loading, and analyzed the frequency characteristics and influence factors for the vibrations induced by the blast loading, the dynamic unloading and the combined effects, respectively. Xu et al.^24^ evaluated the relationship between the frequency and radius of a tunnel based on an engineering case. Zhong et al.^25^ found that the plastic strain of rock mass increases with the increase of duration of vibration. Peng et al.^26^ proposed that the prediction formula for the vibration frequency considering effects of height can better reflect the attenuation of blasting vibration frequency during underground drilling. Álvarez-Vigil et al.^27^ predicted attenuation of the primary frequency of blasting vibration through use of an artificial neural network method. Liu et al.^28^ derived the formula for the attenuation of the primary frequency of blasting vibration in drilling and blasting excavation of the tunnel through dimensional analysis, however, these formulae can only be used in specific cases. It can be seen that most of the current researches are concerned with the influence of blasting on the vibration response characteristics of the excavated tunnel itself, and less involves the analysis of the vibration spectrum characteristics of the surrounding rock of the adjacent tunnel.

Based on theoretical analysis, in the present study we derived an expression for vibration velocity spectra in cutting holes and non-cutting holes during multi-hole blasting. Through use of the dynamic finite element method, response characteristics of vibration frequency spectra in the rock surrounding an adjacent tunnel induced by full-section blasting excavation of a tunnel under the high in-situ stress were studied. Furthermore, velocity spectra in the most greatly affected location (haunch) of the adjacent tunnel under the changed spacing between tunnels and diameter were established and the application range of this theoretical formula in actual engineering was studied. The results provide theoretical and practical guidance for predicting safety in similar deep tunnels during blasting.

## Characteristics of vibration velocity and frequency induced by multi-hole blasting in infinite rock mass

### Equivalent elastic boundary and frequency-domain solutions to blasting load

**Figure 1:**
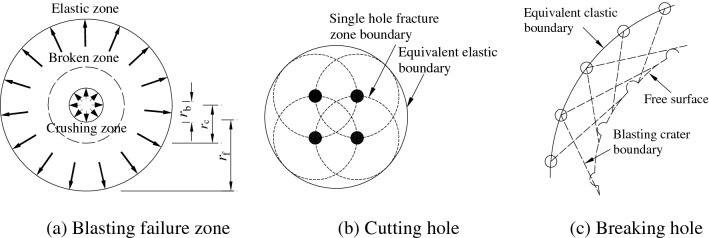
Multiple holes equivalent elastic boundary^31^.

Multi-hole blasting is generally used in blasting excavation of a deep rock mass and it involves the combined blasting of multiple holes in different times and spaces. When geological conditions for blasting-induced stress wave propagation are consistent, the superposition of blasting vibration can be assumed as a linear system^29^. After blasting rock mass, the degree of damage to the rock mass around blast holes can be successively divided into a crushed zone, a fractured zone, and an elastic vibration zone^30^, as shown in Fig. 1a, in which *r*_b_, *r*_c_, and *r*_f_ represent the radius of the blast hole, crushed zone, and fractured zone, respectively. In conventional explosive blasting, *r*_c_ = (3 ~ 5)*r*_b_ and *r*_f_ = (10 ~ 15)*r*_b_. Different blasting conditions in cutting holes and non-cutting holes give rise to differences in equivalent calculation of blasting load. The detonation in a single blast hole can be considered as that of columnar explosives in the semi-infinite medium because the interaction between blasting events is not considered in cutting holes. Therefore, the envelope of the fractured zone caused by detonation in a single hole is an equivalent elastic boundary for detonation in cutting holes and rock mass enclosed by the equivalent elastic boundary is equivalent to a circle of area *A*. The equivalent radius can be calculated based on *r*_e_ = (*A*/π)^1/2^, as shown in Fig. 1b.

Based on the above theory, Wang et al.^32^ derived the peak value of equivalent explosion load in multiple holes blasting.

1 $$P_{b1} = kP_{0} \left( {\frac{{r_{{\text{b}}} }}{{r_{{\text{c}}} }}} \right)^{{2 + \frac{\nu }{1 - \nu }}} \left( {\frac{{r_{{\text{c}}} }}{{r_{{\text{f}}} }}} \right)^{{2 - \frac{\nu }{1 - \nu }}} ;P_{0} = \frac{{\rho_{0} C_{{\text{d}}}^{2} }}{2(\gamma + 1)};k = n\left( {\frac{{r_{{\text{f}}} }}{{r_{{\text{e}}} }}} \right)^{2}$$

where, *P*_0_ is the peak load of single hole blasting under coupling charge, *ρ*_0_ is the initial density of explosive, *C*_d_ is detonation velocity of explosive, *ν* is Poisson's ratio, *n* is the number of holes, *k* is a parameter related to the number and distribution of holes.

However, the blasting process of non-cutting holes is affected by a free face formed by blasting in cutting holes or non-cutting holes in the front segment. Therefore, the line that connects the centers of non-cutting holes and blast holes is regarded as the equivalent elastic boundary, as shown in Fig. 1c. Supposing that blast holes have the same spacing *h* and are arranged regularly, the load can be directly equivalent to that on the excavation profile in the simulation on attenuation of blasting vibration of middle and far zones in non-cutting holes. If the blast source is simplified and does not need to reflect shapes of the blast holes, an influence coefficient *β* of uncoupled charge is introduced because uncoupled charge is generally used in non-cutting holes, the equivalent load to be applied on the excavation profile is expressed as follows^33^:

2 $$P_{b2} = \left( {\frac{{2r_{{\text{f}}} }}{{\text{h}}}} \right)P_{0} \beta ;\;\beta = \left( \frac{a}{b} \right)^{2\gamma }$$

where, *γ* is the isentropic index, which is related to charge density *ρ*_0_, the value of *γ* is 2.1 when *ρ*_0_ < 1200 kg/m^3^, and the value of *γ* is 3.0 when *ρ*_0_ ≥ 1200 kg/m^3^; *a* and *b* are charge diameter and hole diameter respectively.

**Figure 2:**
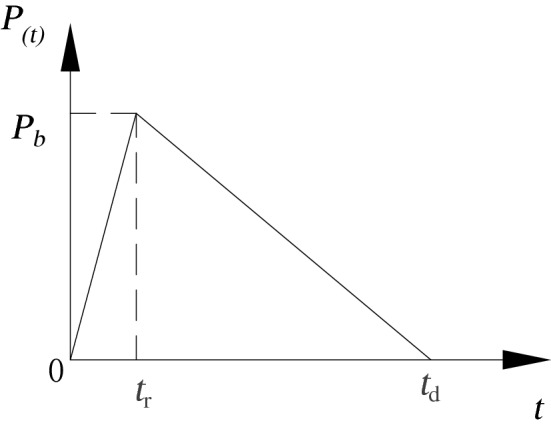
Triangular blasting load curve.

For the time history of load variation in blast holes, the trigonometric function and double exponential function are mainly used in current stages. Finding the coefficient of the double exponential function depends on in-situ test data, so the function is of limited utility and exhibits randomness in practical application. The curve of the trigonometric function of blasting load can simply present the loading and unloading process of blasting vibration waves, as shown in Fig. 2, in which *t*_r_ and *t*_d_ separately indicate the rise time and fall time.

Its time-domain expression is:

3 $$P(t) = \left\{ {\begin{array}{*{20}l} 0 \hfill & {t < - t{\text{r}}} \hfill \\ {P{\text{b}}(1 + t/t{\text{r}})} \hfill & { - t{\text{r}} \le t < 0} \hfill \\ {P{\text{b}}(1 - t/t{\text{d}})} \hfill & {0 \le t < t{\text{d}}} \hfill \\ 0 \hfill & {t > t{\text{d}}} \hfill \\ \end{array} } \right.$$

The rise time of explosion load is the propagation time of detonation wave in the hole, *t*_r_ = *L*_1_/*D.* The duration of explosion load is the time required for the cracks between the boreholes to be connected and the explosive gas to escape until the borehole pressure drops to atmospheric pressure after the explosive is detonated, which can be expressed as^34^:

4 $$t_{S} = \frac{{L_{1} }}{{{\text{C}}_{{\text{d}}} }} + \frac{{\sqrt {\frac{1}{4}h^{2} + L_{2}^{2} } }}{{C_{{\text{f}}} }} + \max \left( {\frac{{L_{1} + L_{2} }}{{V_{{\text{a}}} }},\frac{{L_{1} + L_{2} }}{{C_{{\text{a}}} }}} \right)$$

where, *L*_1_ and *L*_2_ are the length of charge section and plugging section respectively, *C*_f_ is the average velocity of crack expansion driven by explosion load, *C*_a_ is the unloading wave velocity of explosive gas, *V*_a_ is the escape velocity of explosive gas.

Fourier transform was applied to the time-domain expression of triangular blasting load function. Combined with the equivalent peak value calculation formula of blasting load in cutting holes or non-cutting holes group, the amplitude spectrum of triangular load could be obtained as follows:

5 $$AP(\omega ) = \left\{ {1 + a_{e}^{2} + b_{{\text{e}}}^{2} + 2a_{{\text{e}}} b_{{\text{e}}} \cos (\omega t)} \right.\left. { - 2\left[ {a_{{\text{e}}} \cos (b_{{\text{e}}} \omega t) + b_{{\text{e}}} \cos (a_{{\text{e}}} \omega t)} \right]} \right\}^{1/2} \frac{{P_{{{\text{bi}}}} }}{{a_{{\text{e}}} b_{{\text{e}}} t\omega^{2} }}$$

where, *a*_e_ = *t*_r_/*t*, *b*_e_ = *t*_d_/*t*, *P*_bi_ can represent *P*_b1_ or *P*_b2_.

Lu et al.^35^ introduced the medium damping term into the theoretical solution of elastic wave excited by spherical cavity, and obtained the amplitude spectrum of blasting vibration velocity in viscous rock mass:

6 $$F_{{\text{n}}} (\omega ) = \frac{{\exp \left( { - \frac{\omega R}{{2Q_{{\text{r}}} C_{{\text{p}}} }}} \right)A_{{\text{p}}} (\omega )r_{{\text{e}}} C_{{\text{p}}} \omega \sqrt {C_{{\text{p}}}^{2} + R^{2} \omega^{2} } }}{{4\mu r^{2} \sqrt {x + y} }}$$

where, $x = (C_{{\text{p}}} /r_{{\text{e}}} )^{4} + \left( {1 - \frac{\lambda + 2\mu }{{2\mu }}} \right)(C_{{\text{p}}} /r_{{\text{e}}} )^{4} \omega^{2} ,\;\;y = \left( {\frac{\lambda + 2\mu }{{4\mu }}} \right)^{2} \omega^{4}$where, *λ* and *μ* are Lame constants, *C*_p_ is the velocity of longitudinal wave, *Q*_r_ is the geological quality factor of rock, *r*_e_ is the porous equivalent elastic cavity radius, *ω* is angular frequency, *R* is the distance from blasting center.

Combined with Eqs. (1) and (2), the amplitude spectrum of vibration velocity of cutting holes or non-cutting holes group blasting can be obtained as follows:

7 $$F_{n1} (\omega ) = \frac{{\exp \left( { - \frac{\omega r}{{2Q_{r} C_{p} }}} \right)n\left( {\frac{{r_{f} }}{{r_{e} }}} \right)^{2} \left( {\frac{{r_{b} }}{{r_{c} }}} \right)^{{2 + \frac{v}{1 - v}}} \left( {\frac{{r_{c} }}{{r_{f} }}} \right)^{{2 - \frac{v}{1 - v}}} \left( {\frac{{r_{a} }}{{r_{b} }}} \right)^{s\gamma } zP_{0} r_{e} C_{p} \omega \sqrt {C_{p}^{2} + r^{2} \omega^{2} } }}{{a_{e} b_{e} t\omega \cdot 4\mu r^{2} \sqrt {x + y} }}$$

8 $$F_{n2} (\omega ) = \frac{{\exp \left( { - \frac{\omega R}{{2Q_{r} C_{p} }}} \right)4r_{b} r_{f} \left( {\frac{{r_{a} }}{{r_{b} }}} \right)^{s\gamma } zP_{0} r_{e} C_{p} \omega \sqrt {C_{p}^{2} + r^{2} \omega^{2} } }}{{a_{e} b_{e} t\omega hL_{s} \cdot 4\mu r^{2} \sqrt {x + y} }}$$

where, $${\text{z}} = \left\{ {1 + a_{e}^{2} + b_{e}^{2} + 2a_{e} b_{e} \cos (\omega t)} \right.\left. { - 2\left[ {a_{e} \cos (b_{e} \omega t) + b_{e} \cos (a_{e} \omega t)} \right]} \right\}^{1/2}$$.

### Attenuation of the vibration frequency of rock mass induced by multi-hole blasting

The attenuation of the primary vibration frequency in multi-hole blasting was researched by taking detonation in cutting holes and caving holes as an example. By referring to layout of cutting holes in Pubugou Hydropower Station^36^, four-hole cutting blasting was taken as the research object, while the case of other numbers of cutting holes could be deemed analogous to this method. By taking the simultaneous detonation of the outermost caving holes during the full-section blasting excavation as an example, the radius of excavation face was determined to be 3.4 m and the number of blast holes was 20. Assume that the same type of hole is detonating at the same time with the same explosive parameters, the diameter of the hole is 42 mm, the rock parameters *Q*_r_ = 12, *E* = 50 GPa, *G* = 13.5 GPa, *C*_p_ = 4600 m/s, *v* = 0.22, and the blasting load parameters *t*_r_ = 0.7 ms, *t*_d_ = 3.9 ms.

**Figure 3:**
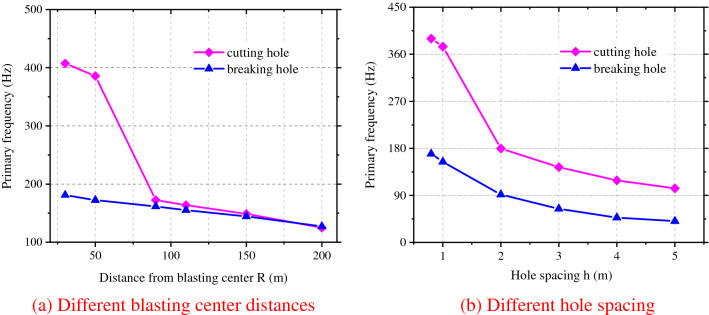
Primary frequency attenuation curve of group hole blasting vibration.

Under a spacing between adjacent blast holes *h* of 0.8 m and the radii of equivalent elastic boundaries of cutting holes and caving holes of 1.3 m and 3.2 m, the attenuation curve of the primary frequency of blasting vibration with the distance from blasting center can be obtained from Fig. 3a: the primary vibration frequency induced by detonation in the two types of blast holes both attenuates with the increase of the distance from blasting center and the primary frequency induced by blasting of cutting holes is greater than that of caving holes under the same distance from blasting center. When the distance from blasting center is less than 90 m, the fundamental frequency mode of vibration attenuates more quickly during detonation in cutting holes, so detonation in caving holes is more likely to cause resonance of rock surrounding compared with detonation in cutting holes, thus damaging the surrounding rock. This is consistent with conclusion of Yi et al.^37^ that the vibration velocity in the surrounding rock induced by detonation in caving holes is larger than that in cutting holes within a certain range in the middle and far zones during blasting excavation of the circular tunnel. However, the vibration frequency in caving holes is smaller than that in cutting holes. When the distance from blasting center exceeds 90 m, attenuations of the primary frequency induced by detonation in the two types of blast holes are consistent.

While the distance from blasting center is *R* = 50 m, the attenuation curve of the primary vibration frequency in multi-hole blasting under different spacings between holes *h* (namely different equivalent elastic boundaries *r*_e_) could be obtained as shown in Fig. 3b. The figure shows that the primary frequency during detonation in the two types of blast holes attenuates with the increase of *h* and the primary frequency attenuates at different velocities due to different spacings between blast holes. During the detonation in cutting holes, the attenuation velocity of the primary frequency with *h* is greater than that in caving holes. Therefore, the detonation in caving holes exerts the greatest influences on vibration of rock surrounding. The blasting in caving holes will be studied in the following work.

## Full-section blasting excavation model of ideal parallel circular tunnels

### Calculation model and parameter design

**Figure 4:**
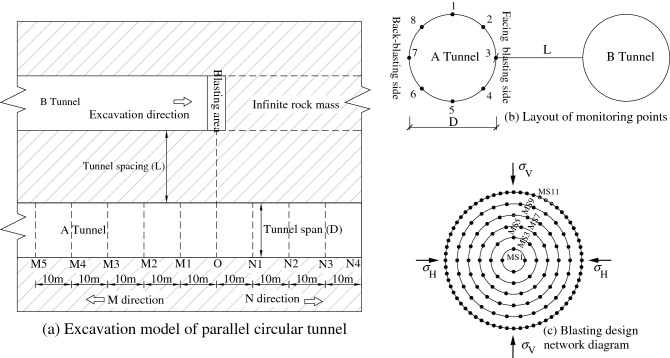
Blasting excavation model and scheme of parallel circular tunnels.

By using Ls-Dyna dynamic finite element analysis method, the blasting excavation process of deep parallel circular tunnels was simulated (Fig. 4a), in which A and B separately denote the adjacent tunnel excavated first and the tunnel to be excavated next. Taking section O as the benchmark, it was stipulated that the M-direction was parallel to the rear of the blasting zone of tunnel A. Sections M1, M2, M3, M4, and M5 were taken along the axial direction of the tunnel along M direction, at distances of 10, 20, 30, 40 and 50 m from section O. The N-direction was parallel to the front of the blasting zone. Sections N1, N2, N3, and N4 at distances of 10, 20, 30, and 40 m from section O were taken along the axial direction of the tunnel in the N-direction. Tunnel A was excavated for 50 m longer than tunnel B, both through full-section excavation and they were circular after excavation.

To study effects of different spatial distributions of tunnels on characteristics of vibration frequency spectra in rock surrounding of the adjacent tunnel, two working conditions were designed as follows: (1) When the span of tunnels was 10 m and location of tunnel B changed, characteristics of blasting vibration frequency spectra in a typical location of the rock surrounding the adjacent tunnel were studied under spacings *L* of 7.5, 10, 12.5, 15, 20, 25, and 30 m between tunnels. (2) Under a spacing of 15 m between tunnels and span of tunnel B of 10 m, characteristics of blasting vibration frequency spectra in the typical location of rock surrounding of the adjacent tunnel were determined under the spans *D* of tunnel A of 5, 10, 15, 20, 25, and 30 m. The layout of measuring points for calculation on each section is shown in Fig. 4b.

There was one circle of cutting holes, three circles of caving holes, one circle of buffer holes, and one circle of smooth blasting holes on the excavation face from inside to outside. They were successively detonated with millisecond-delay detonators in segments of MS1, MS3, MS5, MS7, MS9, and MS11 (Fig. 4c). The emulsified explosives with a density of 1000 kg/m^3^ and detonation wave velocity of 3600 m/s were used for hole-bottom detonation. The characteristics of vibration frequency spectra in the surrounding rock induced by blasting segment-by-segment (MS3, MS5, and MS7) in caving holes were mainly simulated. The ring diameters is 2.2 m, 2.8 m and 3.4 m, and the number of holes in each section is 14, 17 and 20, respectively. The blasting parameters are as follows: the hole diameter is 42 mm, the coil diameter is 32 mm, the hole spacing is 0.8 m, the hole length is 3.0 m, the charging section length is 2.6 m, the blocking section length is 0.4 m, and the single hole charge is 1.6 kg. The distribution of triangular blasting load in Fig. 2 was adopted, where *t*_r_ is 0.7 ms and *t*_d_ is 3.9 ms.

The plastic kinematic hardening model could accurately reveal dynamic response characteristics of rock surrounding under explosion^38^ and in-situ stress of 20 MPa was applied in horizontal and vertical directions. Non-reflecting boundary conditions should be applied on the outer boundary nodes to eliminate influences of reflection of stress waves by free surfaces on results. To ascertain the dynamic response of structures under the blasting load, a modal analysis was necessary to determine the intrinsic frequency and mode of vibration of structures.

### Validation of numerical model

**Figure 5:**
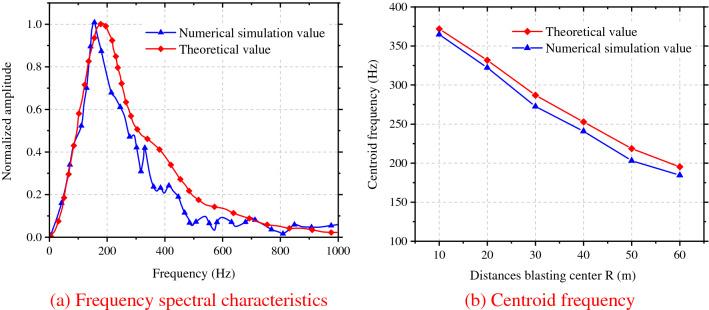
Comparison between theoretical calculation and numerical simulation results.

At the location 30 m from blasting center, the curve of frequency spectral characteristics of blasting-induced seismic waves propagating along the radial direction induced by detonation in the segment MS7 in caving holes with a single blasting footage of 3 m was selected. Through comparisons, it was found to be similar to the results calculated by Formula (8), as shown in Fig. 5a; because the primary frequency identified by Fourier transform alone could not reflect the compositions of the whole blasting vibration frequency spectra, the concept of centroid frequency as shown in Formula (9) was introduced. Figure 5b demonstrates a comparison between theoretical calculation and numerical simulation results of the centroid frequency, with an error of less than 5%. Based on this, the accuracy of numerical simulation was further verified.

9 $$f_{{\text{c}}} = \sum\limits_{{{\text{i}} = 1}}^{{\text{n}}} {A_{{\text{i}}} f_{{\text{i}}} } /\sum\limits_{{{\text{i}} = 1}}^{{\text{n}}} {A_{{\text{i}}} }$$

where, *f*_c_ is the centroid frequency, *A*_i_ is the amplitude spectrum of vibration velocity corresponding to frequency *f*_i_.

## Analysis and discussion on characteristics of vibration velocity and frequency of the rock surrounding the adjacent tunnel induced by blasting excavation

### Influences of changes in spacing between adjacent tunnels

#### Changes of vibration velocity of rock surrounding of the adjacent tunnel under different spacings

**Figure 6:**
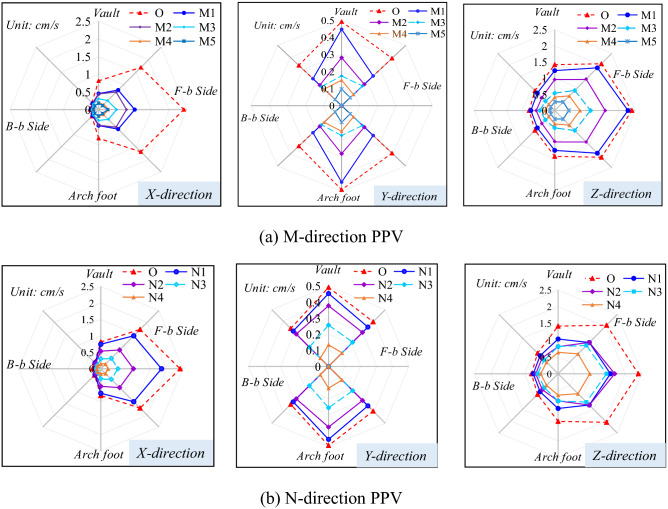
PPV distribution in surrounding rock of adjacent tunnel (*L* = 15 m, *D* = 10 m, F-b Side is the abbreviation for facing blasting side, B-b Side is the abbreviation for back-blasting side).

By taking *L* = 15 m and *D* = 10 m as examples, the distributions of the peak vibration velocity of particles on the wall of tunnel A along the X, Y, and Z-directions under blasting excavation of tunnel B are plotted in Fig. 6. It is stipulated that X, Y and Z directions separately represent the horizontal radial direction, horizontal tangential direction and vertical direction. In the three directions, the vibration velocity of rock mass in the side facing blasting is larger (by 1.1 to 17 times) than that in the back-blasting side, because blasting-induced seismic waves in the former side propagate through diffraction and the intensity decreases gradually. Due to the presence of a free face in the tunnel, rock mass is less confined in the X-direction, so the vibration velocity in the X-direction is the largest. In general, the vibration velocity in the X-direction is taken as the safety criterion for blasting vibration.

By comparing the vibration velocities of each part around the tunnel, the maximum vibration velocity is found at haunch in the side facing blasting (3# measuring point). At the same height as the blasting center, blasting-induced seismic waves are vertically incident, making this the location most significantly affected by blasting vibration: because the tunnel is circular, the angle of incidence of blasting-induced seismic waves increases continuously along the haunch to the vault or the bottom of the arch, so that the input of vibration energy is weakened and the vibration velocity of the surrounding rock decreases from the haunch to vault or bottom of the arch. The vibration velocities at each measuring point of the tunnel reach the maximum on section O and reduce along the M and N-directions with the increase of the distance from blasting center. The attenuation rates of the vibration velocity and frequency of rock mass parallel to the rear of the blasting zone are greater than that in front of the blasting zone, because the existing tunnel parallel to the rear of the blasting zone is less confined by the rock mass.

**Figure 7:**
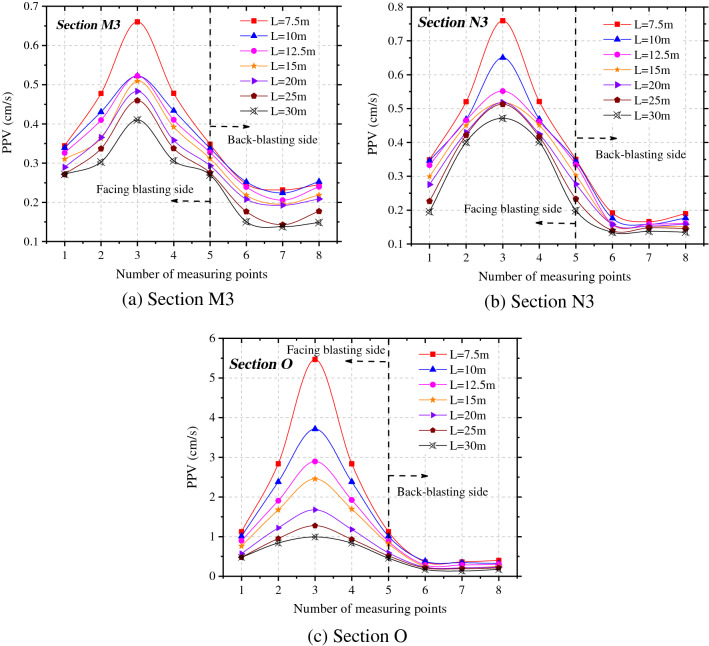
PPV of rock surrounding the adjacent tunnel under different spacings between tunnels.

To investigate the influences of different spacings between tunnels on the peak vibration velocity of rock surrounding the adjacent tunnel, the peak vibration velocities in the X-direction at each measuring point on sections O, M3, and N3 under various working conditions were obtained, as demonstrated in Fig. 7. Under seven different spacings between tunnels, the maximum vibration velocity in X direction of the adjacent tunnel always appears at 3# measuring point (haunch in the side facing blasting). The vibration velocities at each measuring point in the side facing blasting are significantly different, while the vibration velocity of particles in the back-blasting side changes slightly. This is because the intensity of vibration waves attenuates after diffraction, so that the effects in the back-blasting side are much smaller than those in the side facing the blasting. The degree of influence of blasting vibration waves on key parts, namely the arch foot and arch ring in the side facing the blasting, the vault and arch ring, and arch foot in the back-blasting side of the adjacent tunnel are ranked in a descending order.

Figure 7 shows that the smaller the spacing between tunnels is, the larger the vibration velocity of rock surrounding the adjacent tunnel and the lower the safety factor. The vibration velocities in different locations of the adjacent tunnel always decrease with the increase of the spacing between tunnels, while the vibration velocity attenuates at different rates at different measuring points on the same section.

**Figure 8:**
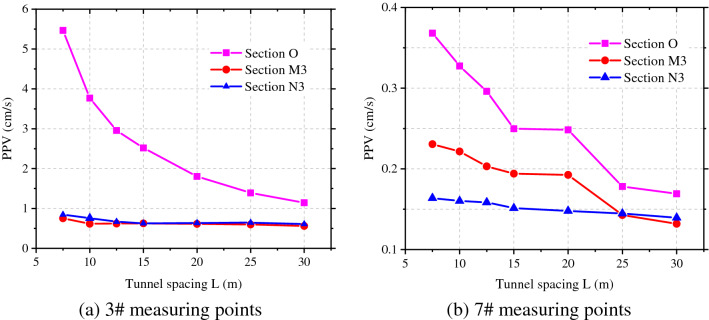
Radial PPV of measuring points 3# and 7# at different tunnel spacings.

By taking measuring points 3 and 7 as examples, the attenuation of the peak vibration velocity in the radial direction of rock surrounding of the adjacent tunnel with the spacing between tunnels was ascertained, as displayed in Fig. 8. The peak vibration velocity at measuring points on section O where the blasting center is located attenuates fastest with the spacing between tunnels, followed by section M3 to the rear of the blasting zone, while the velocity on section N3 attenuates slowest in front of the blasting zone. When the spacing between tunnels is 1.5 times the tunnel span, the attenuation rate of the peak vibration velocity at measuring points on each section with the spacing between tunnels gradually decreases.

The logarithmic transformation of Sadovsky’s vibration formula can be obtained:

10 $$InV = InK + \beta In\left( {\frac{{\sqrt[3]{Q}}}{R}} \right)$$

where, *K* is the coefficient related to rock properties, *β* is the attenuation coefficient, generally *β* = 1 ~ 2, *Q* is a single detonating charge.

When, $y = InV$, $x = In {\frac{{\sqrt[3]{Q}}}{R} }$, $c = InK$, $k = \beta$, there is $y = kx + c$. According to regression analysis, the attenuation of the peak vibration velocity in the side facing the blasting of the adjacent tunnel with the spacing between tunnels was obtained, as shown in Table 1.

**Table 1:** PPV fitting formula of measuring points 3# and 7# at different cavern spacing.

Location	Section O	Section M3	Section N3
3#	$V = 12.76\left( {\frac{{\sqrt[3]{Q}}}{R}} \right)^{1.58} ,{\text{R}}^{2} = 0.{997}$	$V = 2.70\left( {\frac{{\sqrt[3]{Q}}}{R}} \right)^{1.00} ,{\text{R}}^{2} = 0.{984}$	$V = 3.26\left( {\frac{{\sqrt[3]{Q}}}{R}} \right)^{1.05} ,{\text{R}}^{2} = 0.{975}$
7#	$V = 7.96\left( {\frac{{\sqrt[3]{Q}}}{R}} \right)^{2.26} ,{\text{R}}^{2} = 0.{989}$	$V = 3.71\left( {\frac{{\sqrt[3]{Q}}}{R}} \right)^{1.93} ,{\text{R}}^{2} = 0.{99}0$	$V = 2.86\left( {\frac{{\sqrt[3]{Q}}}{R}} \right)^{2.36} ,{\text{R}}^{2} = 0.{996}$

#### Changes of vibration frequency of rock surrounding the adjacent tunnel under different spacings

**Figure 9:**
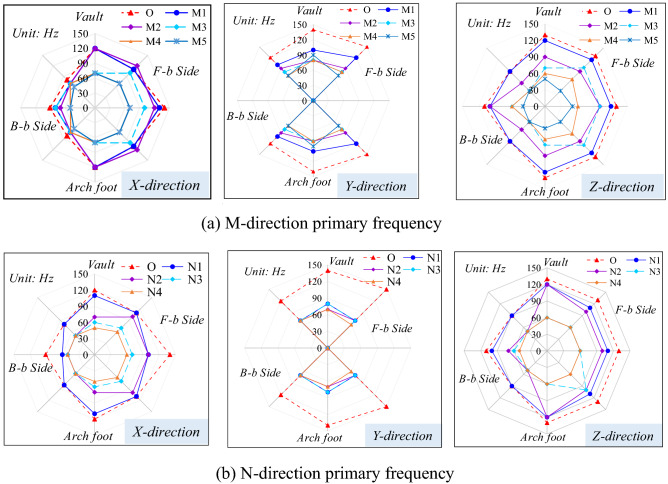
Primary frequency of surrounding rock vibration in adjacent tunnel (*L* = 15 m, *D* = 10 m).

Similarly, considering that the spacing between tunnels *L* = 15 m and *D* = 10 m, Fig. 9 demonstrates changes of the primary vibration frequencies in the X, Y, and Z-directions on each section of the wall of tunnel A.

In the three directions, the primary vibration frequencies of rock surrounding in the side facing the blasting are always greater than those in the back-blasting side. The maximum primary frequency in the X-direction appears in the location where blasting vibration waves are vertically incident, namely the arch foot in the side facing the blasting. The primary vibration frequencies at each measuring point on the tunnel reach their maximum on section O and attenuate to different degrees along the M and N-directions. Moreover, the rates of attenuation change in different directions and at different points. The primary frequencies in three directions of vibration velocity, namely the Y, Z, and X-directions in each location around the tunnel are ranked in descending order. The peak vibration velocity in the X-direction is maximized and the primary frequency reaches a minimum, therefore, the primary frequency in the X-direction can be considered as the safety criterion for blasting vibration of tunnels.

**Figure 10:**
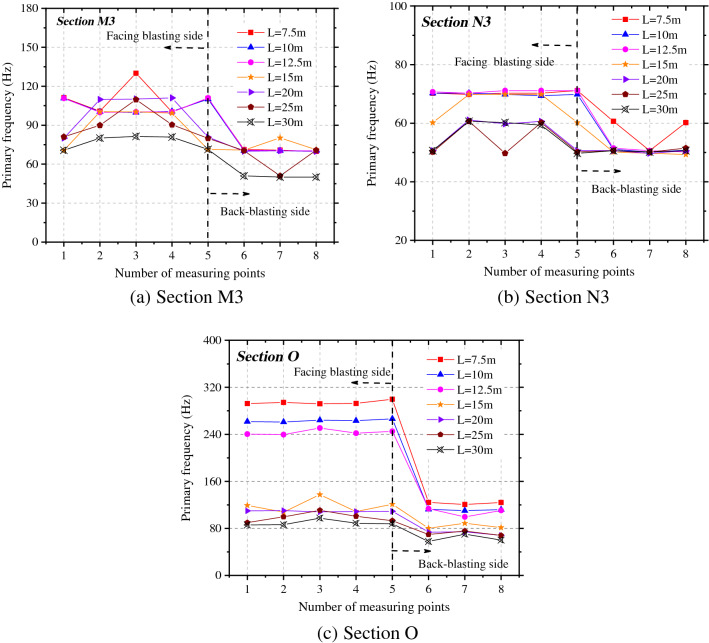
Primary frequency of the adjacent tunnel under different spacings between tunnels.

To explore effects of the different spacings between tunnels on the vibration frequency of rock surrounding of the adjacent tunnel, the changes of the primary frequency in the X-direction at each measuring point on sections O, M3, and N3 were explored, as shown in Fig. 10. Under each working condition, the primary vibration frequency of rock surrounding in the side facing blasting is always greater than that on the back-blasting side. The primary vibration frequencies at each measuring point in rock surrounding the adjacent tunnel attenuate to different degrees with the increase of the spacing between tunnels. The maximum primary frequency appears on the most greatly affected section O. In the axial direction, the attenuation rate of the primary frequency in the side facing blasting with the spacing between tunnels is larger than that in the back-blasting side and the difference in the primary frequency between the two sides reduces with the increase of the spacing between tunnels.

**Figure 11:**
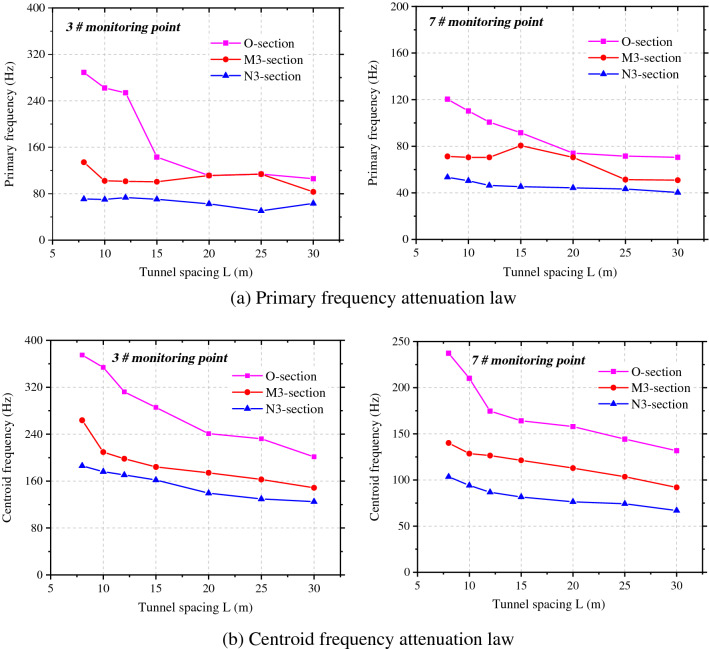
Frequency attenuation law of measuring point 3# and 7# under tunnel spacings.

Figure 11 shows the attenuations of the primary vibration frequency and centroid frequency at the haunches in the side facing blasting and back-blasting side under different spacings between tunnels. The primary vibration frequency does not strictly attenuate with the increasing spacing between tunnels, and there are sudden increase, sudden decrease and small fluctuation in local places. On the most greatly affected section O, the primary vibration frequency and centroid frequency of rock surrounding reach the maximum and the primary frequency attenuates to varying degrees with the tunnel diameter on sections M3 and N3. However, the primary frequency and centroid frequency on section M3 (parallel to the rear of the blasting zone) are greater than those on section N3 (parallel to the front of the blasting zone).

The centroid frequency attenuation law of blasting vibration can be expressed by formula (11).

11 $$f_{c} = \lambda \frac{{C_{p} }}{{Q^{1/3} }} \cdot \left( {\frac{{Q^{1/3} }}{S}} \right)^{\theta } \to f_{c} \cdot \frac{{C_{p} }}{{Q^{1/3} }} = \lambda \cdot \left( {\frac{{Q^{1/3} }}{S}} \right)^{\theta }$$

where, *λ* is a dimensionless parameter, *θ* is the attenuation exponent of blasting vibration frequency with distance.

When, $y = f_{c} \cdot \frac{{C_{p} }}{{Q^{1/3} }}$, $x = \frac{{Q^{1/3} }}{S}$, there is $y = \lambda \cdot x^{\theta }$.

Through regression analysis on the change curve of the centroid frequency in the figure, the attenuations of the centroid frequency at the haunches in the side facing the blasting and back-blasting side with the spacing between tunnels were determined (Table 2).

**Table 2:** *f*_c_ fitting formula of measuring points 3# and 7# at different tunnel spacings.

Location	Section O	Section M	Section N
3#	$f_{c} = 0.75\frac{{C_{p} }}{{Q^{1/3} }} \cdot (\frac{{Q^{1/3} }}{R})^{0.61} ,{\text{R}}^{2} = 0.{985}$	$f_{c} = 2.34\frac{{C_{p} }}{{Q^{1/3} }} \cdot (\frac{{Q^{1/3} }}{R})^{1.30} ,{\text{R}}^{2} = 0.{932}$	$f_{c} = 1.37\frac{{C_{p} }}{{Q^{1/3} }} \cdot (\frac{{Q^{1/3} }}{R})^{1.10} ,{\text{R2}} = 0.{967}$
7#	$f_{c} = 0.93\frac{{C_{p} }}{{Q^{1/3} }} \cdot (\frac{{Q^{1/3} }}{R})^{0.88} ,{\text{R}}^{2} = 0.{99}0$	$f_{c} = 1.93\frac{{C_{p} }}{{Q^{1/3} }} \cdot (\frac{{Q^{1/3} }}{R})^{1.58} ,{\text{R}}^{2} = 0.{986}$	$f_{c} = 0.93\frac{{C_{p} }}{{Q^{1/3} }} \cdot (\frac{{Q^{1/3} }}{R})^{1.99} ,{\text{R}}^{2} = 0.{973}$

### Effects of changes in span of the adjacent tunnel

#### Changes in vibration velocity of rock surrounding of the adjacent tunnel under different spans

**Figure 12:**
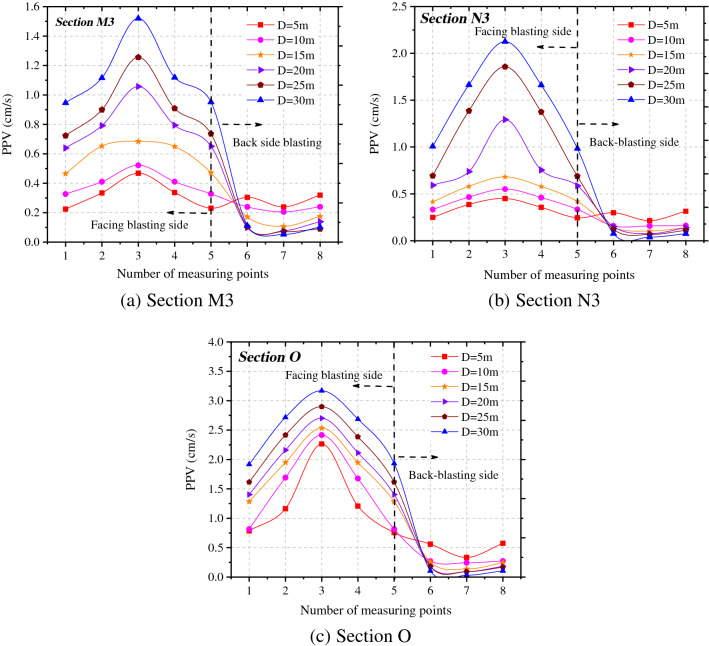
PPV distribution in surrounding rock of adjacent tunnel under different tunnel spans.

Similarly, the peak vibration velocities in the X-direction at each measuring point on sections O, M3, and N3 were analyzed, as shown in Fig. 12. Under the six different tunnel diameters, the maximum vibration velocity in the X-direction of the adjacent tunnel always appears at the 3# measuring point (haunch in the side facing blasting). Overall, the vibration velocities at each measuring point in the side facing the blasting are much larger than those on the back-blasting side. The peak vibration velocity of particles in the side facing the blasting increases with the increase of the tunnel span, while the opposite trend is found in the back-blasting side. The reason for this is that the smaller the tunnel span, the larger the ratio of the length of blasting-induced seismic waves to tunnel diameter. Moreover, most of energy of stress waves diffracts to the back-blasting side, and the reflected energy is attenuated. However, the larger the tunnel span, the smaller the vibration velocity in the back-blasting side because some energy of stress waves is dissipated through reflection.

**Figure 13:**
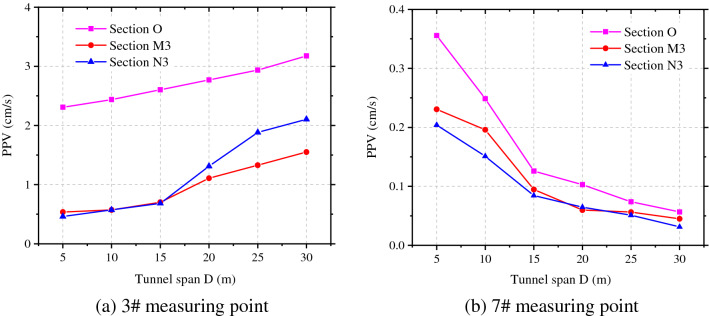
Radial PPV evolution of measuring points 3# and 7# at different tunnel spans.

It can be seen from Fig. 12 that the larger the span of the adjacent tunnel, the higher the vibration velocity of rock surrounding and the smaller the safety factor. To estimate changes of the peak vibration velocities of particles in the side facing the blasting and the back-blasting side, the attenuations of the peak vibration velocities in the X-direction at 3# and 7# measuring points under different tunnel diameters on each section were obtained by measuring at the haunches in both sides, as shown in Fig. 13.

Figure 13 demonstrates that the peak vibration velocity at 3# measuring point (at the haunch in the side facing the blasting) increases with the increase of span of the adjacent tunnel. The peak vibration velocity on the most greatly affected section O is the maximum and it increases at different rates on various sections. The peak vibration velocity at 7# measuring point (haunch in the back-blasting side) on section O where the blasting center is located attenuates fastest with the spacing between tunnels, followed by that on section M3 in rear of the blasting zone, while the smallest attenuation rate is found on section N3 in front of the blasting zone. The curves in the figure were subjected to regression analysis, thus obtaining changes of the peak vibration velocity at the haunch in the side facing blasting of the adjacent tunnel with the tunnel diameter (Table 3).

**Table 3:** *PPV* fitting formula of measuring points 3# and 7# at different tunnel spans.

Location	Section O	Section M3	Section N3
3#	$V = 1.65\left( {\frac{{\sqrt[3]{Q}}}{{R{\text{ + D}}/2}}} \right)^{0.50} ,\;\;{\text{R}}^{2} = 0.{979}$	$V = 1.28\left( {\frac{{\sqrt[3]{Q}}}{{R{\text{ + D}}/2}}} \right)^{0.25} ,\;\;{\text{R}}^{2} = 0.{98}0$	$V = 1.06\left( {\frac{{\sqrt[3]{Q}}}{{R{\text{ + D}}/2}}} \right)^{0.06} ,\;\;{\text{R}}^{2} = 0.{953}$
7#	$V = 2.66\left( {\frac{{\sqrt[3]{Q}}}{R + D}} \right)^{2.26} ,\;\;{\text{R}}^{2} = 0.{95}0$	$V = 694.44\left( {\frac{{\sqrt[3]{Q}}}{R + D}} \right)^{4.66} ,\;\;{\text{R}}^{2} = 0.{978}$	$V = 2500.64\left( {\frac{{\sqrt[3]{Q}}}{R + D}} \right)^{6.28} ,\;\;{\text{R}}^{2} = 0.{899}$

#### Changes of vibration frequency of rock surrounding of the adjacent tunnel under different spans

**Figure 14:**
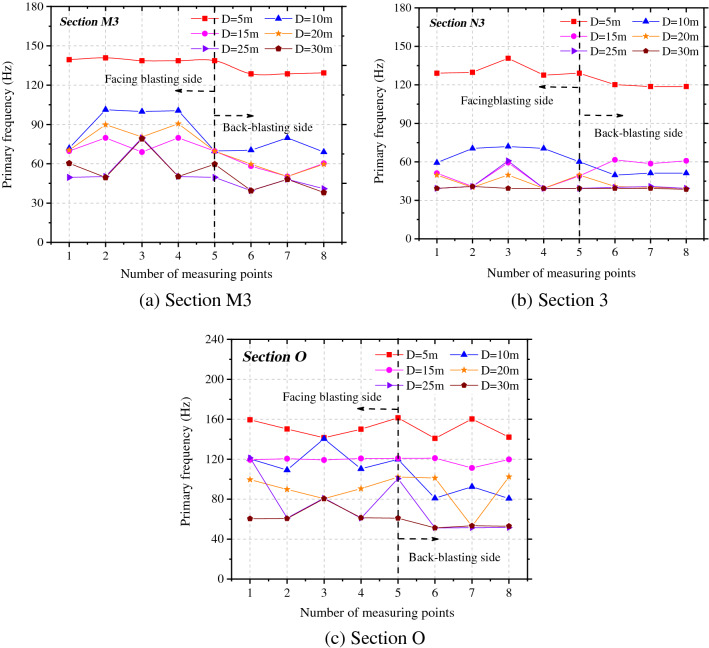
Primary frequency of the adjacent tunnel under different tunnel spans.

The changes of the primary vibration frequencies in the X-direction at each measuring point on sections O, M3, and N3 were evaluated, as illustrated in Fig. 14. Under each working condition, the primary vibration frequency of the rock surrounding the side facing the blasting is slightly different from that on the back-blasting side. The primary vibration frequencies of rock surrounding at each measuring point of the adjacent tunnel attenuate to different degrees with the enlargement of the tunnel diameter. In different axial locations in the surrounding rock, the primary vibration frequencies of particles attenuate at different velocities along the M and N-directions.

**Figure 15:**
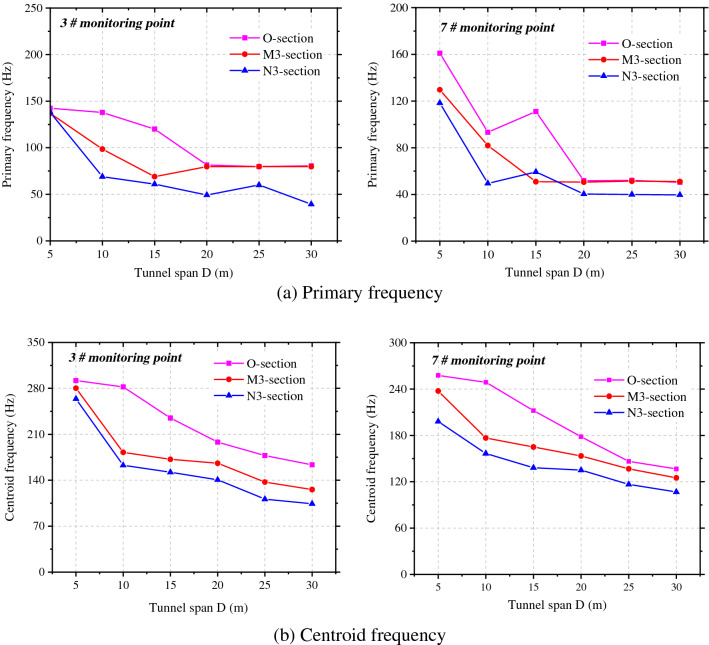
Frequency attenuation law of measuring point 3# and 7# under different tunnel spans.

Figure 15 illustrates the attenuations of the primary vibration frequency and centroid frequency at haunches in the side facing the blasting and back-blasting side under different spacings between tunnels. The primary vibration frequency of rock surrounding attenuates overall with the increase of the tunnel diameter, while it fluctuates slightly in some locations but does not attenuate in a strictly monotonic manner. On the most significantly affected section (section O), the primary vibration frequency and centroid frequency of rock surrounding are maximized. The primary frequency attenuates to varying degrees with the tunnel diameter on sections M3 and N3, while the primary frequency and centroid frequency on section M3 are larger than those on section N3.

The regression analysis was performed on change curves of the centroid frequency at haunches in the side facing the blasting and back-blasting side in the figure. The attenuation of the centroid frequency in the side facing the blasting of the adjacent tunnel with the tunnel diameter is attained through fitting, as shown in Table 4.

**Table 4:** *f*_c_ fitting formula of measuring points 3# and 7# at different tunnel spans.

Location	Section O	Section M3	Section N3
3#	$f_{c} = 2.35\frac{{C_{p} }}{{Q^{1/3} }}\left( {\frac{{Q^{1/3} }}{{R{\text{ + D}}/2}}} \right)^{1.46} ,\;\;{\text{R}}^{2} = 0.{967}$	$f_{c} = 108.14\frac{{C_{p} }}{{Q^{1/3} }}\left( {\frac{{Q^{1/3} }}{{R{\text{ + D}}/2}}} \right)^{3.42} ,\;\;{\text{R}}^{2} = 0.{877}$	$f_{c} = 314.45\frac{{C_{p} }}{{Q^{1/3} }}\left( {\frac{{Q^{1/3} }}{{R{\text{ + D}}/2}}} \right)^{4.10} ,\;\;{\text{R}}^{2} = 0.{886}$
7#	$f_{c} = 2.12\frac{{C_{p} }}{{Q^{1/3} }}\left( {\frac{{Q^{1/3} }}{{R{\text{ + D}}/2}}} \right)^{1.89} ,\;\;{\text{R}}^{2} = 0.{983}$	$f_{c} = 99.6\frac{{C_{p} }}{{Q^{1/3} }}\left( {\frac{{Q^{1/3} }}{{R{\text{ + D}}/2}}} \right)^{2.99} ,\;\;{\text{R}}^{2} = 0.{896}$	$f_{c} = 267.3\frac{{C_{p} }}{{Q^{1/3} }}\left( {\frac{{Q^{1/3} }}{{R{\text{ + D}}/2}}} \right)^{3.36} ,\;\;{\text{R}}^{2} = 0.{9}0{1}$

### Vibration velocity spectra in rock surrounding the adjacent circular tunnel

Based on the above analysis, it was found that the blasting vibration waves exert the greatest influences on the haunch in the side facing the blasting of the adjacent tunnel, where the vibration velocity is inversely proportional to the spacing between tunnels and directly proportional to the tunnel diameter. The centroid frequency increases with the increase of the spacing between tunnels and tunnel diameter and the most greatly affected section is that where the excavation face is located. Based on changes of the vibration velocity and frequency of rock surrounding under different spacings between tunnels and tunnel diameters in Tables 2, 3, and 4, the relationships of the spacing between tunnels and tunnel diameter with peak particle velocity (PPV) and frequency were explored as follows:

12 $$V^{\prime} = V\left( {\frac{{Q^{1/3} }}{{L + R_{D} }}} \right)^{1.58\eta } ;\;\;f_{c}^{\prime } = f_{c} \left( {\frac{{Q^{1/3} }}{{L + R_{D} }}} \right)^{0.61\eta }$$

13 $$V^{\prime\prime} = V\left( {\frac{{Q^{1/3} }}{{{\text{R}} + {\text{D/2}}}}} \right)^{0.50\zeta } ;\;\;f_{c}^{\prime \prime } = f_{c} \left( {\frac{{Q^{1/3} }}{{{\text{R}} + {\text{D/2}}}}} \right)^{1.46\zeta }$$

where, *η* is the influence coefficient of cavity spacing change, when *L* = 0, *η* = 0, in other cases, *η* = 1; *ζ* is the influence coefficient of cavity span change, when *D* = 0, *ζ* = 0, and in other cases, *ζ* = 1.

On the basis of Formula (8) and in combination with the above rules, the velocity amplitude spectrum at the haunch in the side facing the blasting of the adjacent tunnel when the spacing of cavities or the diameter of cavities changes can be deduced as follows:

14 $$F_{{n_{2} }}^{\prime } (\omega^{\prime})_{{R = R_{D} }} = F_{{n_{2} }} (\omega^{\prime}) \cdot \left( {\frac{{(n\pi r_{a}^{2} L_{1} )^{1/3} }}{{L + R_{D} }}} \right)^{0.61\eta } ;\;\;\omega^{\prime}{ = }\omega \cdot \left( {\frac{{(n\pi r_{a}^{2} L_{1} )^{1/3} }}{{L + R_{D} }}} \right)^{1.58\eta }$$

15 $$F_{{n_{2} }}^{\prime \prime } (\omega^{\prime\prime})_{{R = R_{D} }} = F_{{n_{2} }} (\omega^{\prime\prime}) \cdot \left( {\frac{{(n\pi r_{a}^{2} L_{1} )^{1/3} }}{{L + R_{D} { + }\frac{D}{2}}}} \right)^{1.46\zeta } ;\;\;\omega^{\prime\prime}{ = }\omega \cdot \left( {\frac{{(n\pi r_{a}^{2} L_{1} )^{1/3} }}{{L + R_{D} { + }\frac{D}{2}}}} \right)^{0.5\zeta }$$

Equations (14) and (15) are derived on the basis of full section excavation of ideal circular parallel cavern. The research object is surrounding rock vibration induced by initiation of single ring caving hole, and the amplitude and frequency of vibration velocity at the haunch in the side facing the blasting on the most unfavorable section of adjacent cavern can be calculated.

## Application of the formula for velocity spectra in engineering practice

### Blasting test of diversion tunnels in Pubugou Hydropower station

In Pubugou Hydropower Station, water resources were developed with water diversion and power generation and the underground powerhouse tunnels mainly include a main powerhouse, a main transformer chamber, a tailrace sluice chamber, diversion tunnels and tailrace tunnels. There were six diversion tunnels in the diversion system of the Pubugou project and pressure pipes were arranged in parallel, with a center-to-center spacing of 28.86 m and a net spacing of 17.76 m. The section was circular and the inner diameter for excavation was 10.70 to 11.10 m. The longitudinal wave velocity, elastic modulus, density, and Poisson’s ratio of the rock mass were 4500 to 5000 m/s, 47.2 GPa, 2700 kg/m^3^, and 0.23, respectively. The in-situ stress field in the blasting zone was mainly dominated by tectonic stress and the in-situ stresses in horizontal and vertical directions were about 20 MPa, at the medium to high in-situ stresses.

**Figure 16:**
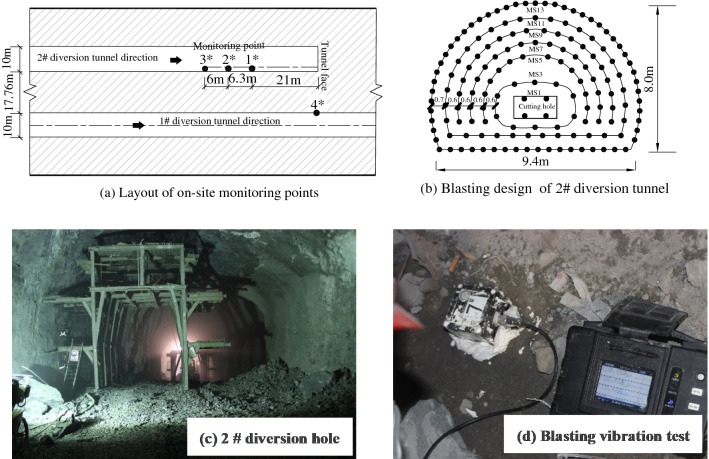
Blasting vibration test of diversion tunnel group of Fubugou Hydropower Station.

The blasting vibration in a certain blasting operation in the 2# tunnel was monitored and measuring points 1*, 2*, and 3* were arranged in rock mass (haunch) of tunnel wall in the rear of the tunnel face for blasting excavation, with distances from the tunnel face of 21.0, 27.3, and 33.3 m. Measuring point 4* was arranged in the existing 1# tunnel adjacent to the 2# tunnel to monitor vibration of the rock surrounding the adjacent tunnel, being 17.76 m from the excavation face. The layout of the measuring points is shown in Fig. 16a. Full-section excavation blasting was adopted in this blasting operation, and the dimensions of the excavation section of the middle drift were 8.0 m × 9.4 m. The blasting design network is displayed in Fig. 16b. Taking caving holes MS5, MS7 and MS9 as research objects, and ring diameters is 2.2 m, 2.8 m and 3.4 m, and the number of holes in each section is 14, 17 and 20, respectively. On-site blasting parameters: the hole length is 1.5 m, the hole diameter is 45 mm, the charge diameter is 32 mm, and the single hole charge is 0.6 kg.

### Field test and validation of calculated results

**Figure 17:**
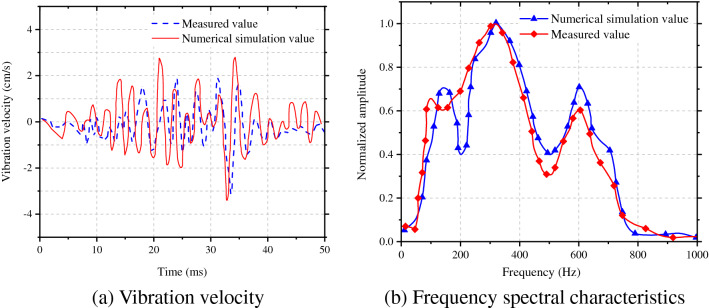
Comparing the measured results and numerical calculation results.

The field test and numerical calculation results at measuring point 1* were taken as examples. The time-history curve of blasting vibration in the horizontal radial direction of the segment MS9 of caving holes was obtained. To facilitate observation and comparison, a waveform of only 50-ms duration (including a peak) was intercepted, as shown in Fig. 17a.

Figure 17b demonstrates the vibration velocity spectra obtained by Fourier transform of the time-history curve of vibration. By comparing the measured results and numerical calculation results of time-history curve and frequency spectra, the results are seen to be consistent, which further verifies the accuracy of the numerical model. Due to influences of factors, such as layout of blast holes, on-site rock mass conditions and layout of measuring points in engineering practice, the velocity and frequency obtained through the numerical simulation are slightly larger than those in practice, while they do not influence the study of changes in velocity and frequency.

### Discussion on the goodness of fit of the theoretical formula

**Figure 18:**
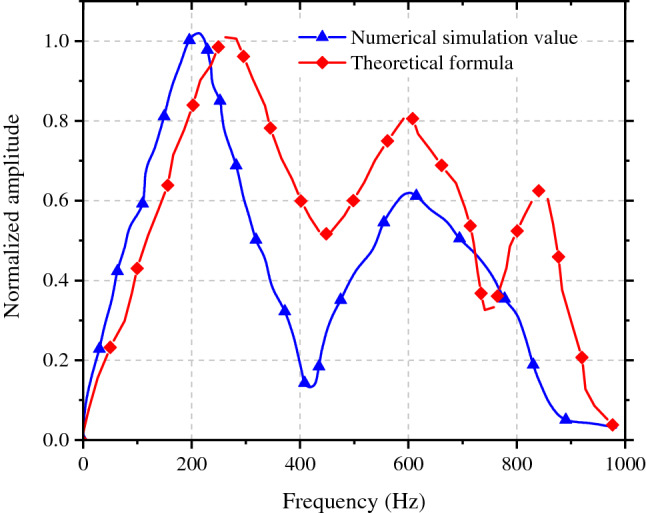
The spectrum curves of surrounding rock on facing blasting side of tunnel.

Based on Formulae (14) and (15), the vibration velocity and frequency of rock surrounding in the side facing the blasting (at the haunch) of the 1# tunnel (adjacent tunnel) were calculated. The vibration velocity spectra in rock surrounding in the side facing the blasting of the 1# tunnel under different spacings between tunnels and tunnel diameters were calculated. The frequency spectra obtained through numerical simulation under the spacing between tunnels of 18 m and span of the adjacent tunnel of 10 m was compared with the theoretical formula, as displayed in Fig. 18.

The primary frequency and average frequency of primary frequency bands in two results are slightly different and frequency spectra show differences in structures. The frequency spectra calculated by the theoretical formula presents a three-peak structure, and that obtained by the numerical simulation presents a two-peak structure. The frequency spectra of the adjacent tunnel exhibit a slightly larger error in the comparison of these results with the tunnel, probably because stress concentration occurs on the wall of the adjacent tunnel, which is not taken into account by the formula for the frequency spectra.

**Figure 19:**
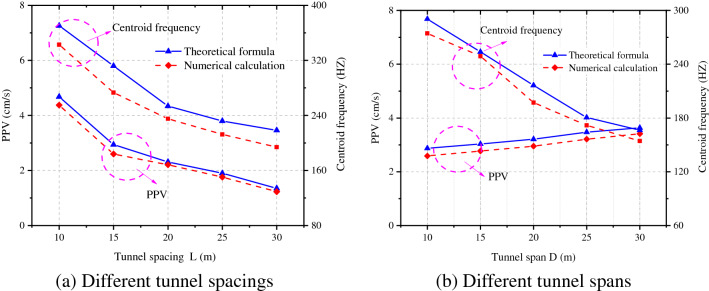
PPV and centroid frequency under different spacings and spans.

Based on the frequency spectra at each point, the attenuations of the vibration velocity and centroid frequency with the spacing between tunnels and tunnel diameter could be obtained and compared with the numerical simulation results, as shown in Fig. 19a and b. The attenuations of the vibration velocity and centroid frequency of rock surrounding of the adjacent tunnel are similar and the results obtained by the theoretical formula are slightly larger than those obtained through numerical simulation. The error in the vibration velocity and frequency under the same working condition ranges from 5 to 12%; because actual blasting excavation can damage the rock surrounding, and between, two tunnels to some extent, the attenuation of velocity and frequency increases in the propagation of blasting-induced seismic waves to the rock surrounding the adjacent tunnel, making the numerical simulation results are lower.

In conclusion, in the application of the expression for vibration velocity spectra derived theoretically in the 2# diversion tunnel of Pubugou Hydropower Station, attenuations of vibration velocity and frequency match those measured during blasting, with small errors in the specific values. This is due to complex conditions, such as layout of blast holes, on-site rock mass conditions and layout of measuring points in engineering practice. The theoretical formula is derived from a circular tunnel excavated in full section in an idealized infinite rock mass, but it provides certain guidance when studying the trends in vibration frequency spectra in the surrounding rock.

## Conclusions

By means of theoretical derivation, dynamic finite element calculation and field measurement, the response characteristics of vibration frequency spectra in rock surrounding an adjacent tunnel induced by full-section blasting excavation of the tunnel under the high in-situ stress were studied. The main conclusions are as follows:

1. Through a theoretical analysis, the frequency-domain expression for the equivalent blasting load in multiple holes was derived. Based on blasting vibration velocity spectra in cohesive rock mass, expressions for vibration velocity spectra of cutting and non-cutting holes in multi-hole blasting operations were obtained. Moreover, it was verified that the vibration velocity in surrounding rock induced by detonation in caving holes was higher than that in cutting holes within a certain range of middle and far zones during blasting excavation of the circular tunnel.
2. The blasting vibration waves show the largest influences on the adjacent tunnel at the haunch in the side facing the blasting, where the vibration velocity is inversely proportional to the spacing between tunnels, while it directly proportional to the tunnel diameter. The centroid frequency increases with the increase of the spacing between tunnels and tunnel diameter and the section most affected by blasting vibration in the adjacent tunnel is the section in which the excavation face is located.
3. Vibration velocity spectra in the most affected location (the haunch) in the side facing the blasting of the adjacent tunnel under changing spacing between tunnels and tunnel spans were determined based on calculations using the full-section excavation model in the ideal parallel circular tunnels. By comparing data with measured blasting data recorded in diversion tunnels in Pubugou Hydropower Station, the goodness of fit of the theoretical formula was verified and the range of application of the theoretical formula for velocity spectra was revealed.

It should be pointed out that the theoretical formula is derived from a circular tunnel excavated in full section in an idealized infinite rock mass, and some simplified methods and semi-empirical formulas are adopted in the numerical simulation to improve the calculation efficiency. Notwithstanding its limitation, the work in this paper still provides some instructive views for the evaluation of the rock vibration generated in blasting excavation of deep tunnels.

## Abbreviations

PPV: Peak particle velocity (cm/s)
*P*_*0*_: Peak load of single hole blasting under coupling charge (Mpa)
*ρ*_0_: Detonation velocity of explosive (m/s)
*h*: Blast holes spacing (m)
*t*_r_: The rise time of explosion load (ms)
*t*_d_: The decline time of explosion load (ms)
*t*_s_: The duration of explosion load (ms)
*ν*: Poisson's ratio
*n*: Number of holes
*k*: A parameter related to the number and distribution of holes
*r*_b_: Radius of the blast hole (m)
*r*_c_: Radius of crushed zone (m)
*r*_f_: Radius of fractured zone (m)
*γ*: Isentropic index
*a*: Charge diameter (mm)
*b*: Hole diameter (mm)
*L*_1_: Length of charge section (m)
*L*_*2*_: Length of plugging section (m)
*C*_f_: The average velocity of crack expansion driven by explosion load (m/s)
*C*_a_: The unloading wave velocity of explosive gas (m/s)
*C*_p_: The velocity of longitudinal wave (m/s)
*V*_a_: The escape velocity of explosive gas (m/s)
*λ*, *μ*: Lame constants
*Q*_r_: The geological quality factor of rock
*r*_e_: The porous equivalent elastic cavity radius (mm)
*ω*: Angular frequency
*R*: The distance from blasting center (m)
*f*_c_: The centroid frequency (Hz)
*A*_i_: The amplitude spectrum of vibration velocity
*K*: The coefficient related to rock properties
*β*: The attenuation coefficient
*Q*: Single detonating charge (kg)
*λ*: A dimensionless parameter
*θ*: The attenuation exponent of blasting vibration frequency with distance
*η*: The influence coefficient of cavity spacing change
*ζ*: The influence coefficient of cavity span change
